# Association of Night Eating Syndrome Risk with Sleep Disturbance in Medical Students: A Cross-Sectional Study

**DOI:** 10.3390/healthcare14162666

**Published:** 2026-08-21

**Authors:** Volkan Tekin, Sümeyra Delibaş, Eyüp Sina Keten, Mustafa Şahan, Mehmet Koçer, Sinan Yetkin

**Affiliations:** 1 Department of Physiology, Gülhane Faculty of Medicine, University of Health Sciences, Ankara 06100, Türkiye; 2 Gülhane Faculty of Medicine, University of Health Sciences, Ankara 06100, Türkiye; 3 Psychiatry Clinic, Gülhane Education and Research Hospital, Ankara 06100, Türkiye

**Keywords:** night eating syndrome, sleep quality, medical students, insomnia, emotional eating, restless legs syndrome, cross-sectional study

## Abstract

**Background:** Medical students are vulnerable to sleep disturbance and nocturnal eating behaviors that may jointly increase psychiatric and metabolic morbidity. Comprehensive sleep-eating evaluations in Turkish medical students remain limited. To determine the prevalence of poor sleep quality and night eating syndrome (NES) risk and to examine their cross-sectional associations with insomnia, anxiety, emotional eating, restless legs symptoms, chronotype, and lifestyle factors. **Methods:** This cross-sectional study, conducted from March to December 2023, included 265 medical students who completed validated instruments assessing sleep quality (Pittsburgh Sleep Quality Index [PSQI]), night eating syndrome (NES) risk (a score of ≥25 indicating high risk), insomnia severity, daytime sleepiness (Epworth Sleepiness Scale [ESS]), anxiety, emotional eating, Restless Legs Syndrome (RLS) symptoms, and chronotype. Group comparisons, Spearman’s correlation analyses, and multiple linear regression were conducted. **Results:** Poor sleep quality (PSQI > 5) affected 50.2% of participants, and high NES risk affected 14.7%. PSQI correlated with NEQ (r = 0.301; *p* < 0.001), insomnia severity (r = 0.538), anxiety, and emotional eating. Mean NEQ was higher in the poor-sleep group (18.67 ± 8.00 vs. 15.38 ± 6.36; *p* < 0.001; Cohen’s d = 0.455). Females had higher PSQI and ESS scores, anxiety, and emotional eating scores; high NES risk was numerically more frequent in males (18.6% vs. 11.8%; *p* = 0.175). RLS symptoms (score > 0) were present in 34.7% (mild 22.6%; moderate 7.9%; severe 4.2%). In the primary multivariable model (PSQI and sex), PSQI was the strongest independent correlate of NEQ (standardized β = 0.325; *p* < 0.001); sensitivity analyses showed stable PSQI estimates after additional covariate adjustment, whereas sex coefficients were non-significant and unstable across models. A secondary model excluding PSQI identified alcohol use as a significant lifestyle correlate (standardized β = 0.167; *p* = 0.014). R^2^ = 0.148 for the full covariate model and R^2^ = 0.108 for the primary model. **Conclusions:** Sleep disturbance and NES risk are prevalent and cross-sectionally associated in Turkish medical students. Poor sleep quality was the strongest independent correlate of night eating scores. Integrated campus screening targeting sleep, eating behavior, anxiety, and comorbid sleep symptoms may facilitate prevention and referral.

## 1. Introduction

Nocturnal eating disorders—including sleep-related eating disorder (SRED) and night eating syndrome (NES)—represent clinically distinct yet overlapping conditions at the interface of sleep medicine, psychiatry, and metabolic health [1].

University students are particularly susceptible to sleep disturbances; a meta-analysis of African university students reported a pooled prevalence of poor sleep quality at 63.3% [2], whereas global medical student data suggest comparably high rates [3]. During periods of academic examinations, perceived stress concurrently exacerbates insomnia and disordered eating behaviors [4]. Sleep restriction decreases leptin levels, increases ghrelin levels, and amplifies hunger, thereby impairing the regulation of appetite [5].

Mechanistic reviews emphasize reciprocal pathways linking sleep disturbance to eating pathology through circadian misalignment and neuroendocrine dysregulation [6]. A recent meta-analysis confirmed associations between disordered eating behaviors and sleep parameters within non-clinical populations [7]. NES is characterized by a delayed circadian timing of food intake relative to sleep [8], whereas bidirectional relationships between insomnia and eating-disorder pathology are increasingly supported [9]. In university cohorts, NES prevalence and subsyndromal night eating features are common [10,11].

Among Turkish university students, poor sleep quality has been associated with disordered eating behavior [12], unfavorable eating attitudes [13], and maladaptive food-related behaviors [14]. In female health sciences students, NES symptoms relate to sleep impairment [15], and poor sleep, emotional eating, and reduced mental well-being frequently cluster in student populations [16]. Evening chronotype is associated with night eating symptoms, partly mediated by depressive affect [17], and the COVID-19 pandemic worsened sleep quality and NES burden among Turkish university students [18].

Despite this body of evidence, only a limited number of studies have concurrently assessed sleep quality, insomnia severity, daytime sleepiness (ESS), NES screening, anxiety, emotional eating, RLS symptoms, and chronotype in a single cohort of Turkish medical students using validated instruments.

Restless Legs Syndrome (RLS) symptoms were additionally evaluated because RLS is a sleep-related sensorimotor disorder frequently associated with sleep fragmentation, insomnia, and circadian/sleep–wake disruption [19,20]. Nocturnal eating may occur as a non-motor manifestation of RLS and has been reported in association with both night eating syndrome and sleep-related eating behaviors [1,19,21]. Although direct evidence linking RLS to NES in student populations remains limited, prior work suggests overlap between restless legs symptoms, poor sleep quality, and disordered eating features [21]. Accordingly, RLS was included as a comorbid sleep-related variable to examine whether night eating scores relate specifically to NES-related sleep features or to broader sleep comorbidity.

Consequently, a cross-sectional study was conducted among medical students at Gülhane Faculty of Medicine. The objectives included estimating the prevalence of inadequate sleep quality and elevated NES risk, characterizing cross-sectional associations between sleep, eating, and psychological variables, and identifying independent correlates of night eating behavior to guide comprehensive campus screening initiatives.

## 2. Materials and Methods

### 2.1. Study Design and Setting

This cross-sectional observational study was conducted at the University of Health Sciences Gülhane Faculty of Medicine in Ankara, Türkiye, from 1 March 2023 to 31 December 2023. The study protocol received approval from the Gülhane Scientific Research Ethics Committee (decision no. 2023-72). Written informed consent was secured from all participants prior to data collection. The study is reported in accordance with the STROBE statement for cross-sectional studies as shown in Table S1 [22].

### 2.2. Participants

Medical students were recruited through voluntary participation via classroom announcements and electronic survey distribution. Inclusion criteria included enrollment at Gülhane Faculty of Medicine, age of 18 years or older, and the completion of all questionnaires. Exclusion criteria encompassed refusal to provide informed consent, incomplete questionnaires, known severe neurological diseases (such as dementia and Parkinson’s disease), and untreated thyroid disorders. No formal a priori sample-size or power calculation was performed. Instead, a minimum recruitment target of 300 volunteers was prespecified in the ethics protocol as a feasibility-oriented enrollment goal for a single-center medical student survey to be completed within one academic year (March–December 2023). This target reflected anticipated accessibility of the student population rather than a statistically derived sample-size requirement for a specific effect size. Of the 300 students who were approached or self-enrolled, 265 (88.3%) provided complete datasets and were included in the analyses; 35 were excluded because of incomplete questionnaires or withdrawal of consent.

### 2.3. Data Collection Instruments

Participants completed a sociodemographic form recording age, sex, body mass index, and binary self-reported history of psychiatric disorder, chronic medical condition, regular medication use, smoking, and alcohol consumption. Psychiatric diagnoses, medication names, and therapeutic classes were not recorded in structured detail, and disease status was not verified against medical records. Participants also completed the following validated instruments: (1) a five-item SRED screening module adapted from the literature [1,23] and administered within the electronic survey to assess nocturnal eating with amnesia or hazardous behavior features; items were translated into Turkish by the study team using a forward-translation procedure. SRED responses were not incorporated into the final merged analytic dataset and are therefore not reported in the primary analysis (see Section 4); (2) Night Eating Questionnaire (NEQ; score ≥25 indicates high NES risk). The Turkish NEQ form validated by Atasoy et al. [24] in an outpatient psychiatric population was used in this study. Although that validation study proposed a lower clinical cutoff (≥18), we applied the ≥25 threshold recommended by Runfola et al. [10] for university student screening to facilitate comparison with the international student literature [10,11]; (3) Pittsburgh Sleep Quality Index (PSQI; Turkish validation PUKI; score >5 indicates poor sleep quality) [25]; (4) Insomnia Severity Index (ISI) with Turkish psychometric support [26]; (5) Epworth Sleepiness Scale (ESS) [27], with Turkish validation [28]; (6) Hamilton Anxiety Rating Scale (HAM-A) [29], administered as a structured clinician-led interview using the validated Turkish form [30]; (7) Turkish Emotional Eating Scale [31], with tension-related and negative-emotion subscale scores used in analyses; (8) Restless Legs Syndrome questionnaire (total score 0–30: 1–10 mild; 11–20 moderate; 21–30 severe) [32]; and (9) Morningness–Eveningness Questionnaire (MEQ). The validated Turkish version [33] was used, with chronotype categories classified according to the thresholds reported in the Turkish validation study [33]: evening type (score ≤ 41), intermediate type (42–58), and morning type (≥59). The original MEQ [34] is cited as the source instrument.

Hamilton Anxiety Rating Scale (HAM-A) administration. Although the self-report questionnaires were distributed electronically, the HAM-A was not self-administered. All 265 participants included in the final analysis underwent a structured clinician-led HAM-A interview conducted by trained evaluators from the Psychiatry Clinic at Gülhane Education and Research Hospital, University of Health Sciences. Evaluators were familiar with the validated Turkish HAM-A form [30] and applied a standardized interview protocol during data collection (March–December 2023). Interviews were conducted in person at the psychiatry clinic after electronic completion of the remaining questionnaires. HAM-A scores were recorded in the study database and merged with questionnaire data using participant identifiers.

### 2.4. Statistical Analysis

Analyses were performed using SPSS 25.0 (IBM, Armonk, NY, USA) and Python 3.14 (Python Software Foundation, Wilmington, DE, USA). Continuous variables are presented as mean ± standard deviation; categorical variables are expressed as *n* (%). Normality was assessed with the Shapiro–Wilk test; non-parametric tests were used when appropriate. Group comparisons were performed using the Mann–Whitney U test for continuous variables and the chi-square test for categorical variables. Spearman’s rank correlation was applied to assess associations among continuous variables. Given that 15 correlation coefficients were tested in the primary correlation matrix, results were cautiously interpreted considering the issue of multiple comparisons; nominally significant associations (*p* < 0.05) are reported without Bonferroni correction, in accordance with exploratory observational analyses. Multiple linear regression analyses were conducted with NEQ score as the dependent variable. Smoking and alcohol use were coded as binary indicator variables (1 = current use, 0 = no use). The primary model included PSQI and sex (female = 1) to minimize multicollinearity and to prioritize the prespecified sleep-eating association. Preplanned sensitivity models sequentially added smoking and alcohol use, and then age and BMI. A secondary model excluding PSQI was also fitted to evaluate lifestyle correlates of night eating independent of sleep quality. Because age and BMI were strongly correlated with sex in this student sample, their simultaneous inclusion with sex can inflate VIF values; therefore, coefficient stability for PSQI and sex was examined across the primary and sensitivity specifications. Variance inflation factors (VIFs) were calculated for the full-covariate model. When VIF exceeded 10, the corresponding regression coefficient was not interpreted inferentially. Stability of the PSQI and sex associations was evaluated using the primary and sensitivity models. Statistical significance was established at *p* < 0.05.

## 3. Results

### 3.1. Participant Characteristics

A total of 265 medical students were included in the analysis. The sample comprised 152 females (57.4%) and 113 males (42.6%), with a mean age of 21.0 ± 1.7 years and a mean BMI of 22.48 ± 3.30 kg/m^2^. Smoking and alcohol use were each reported by 26 participants (9.8%). Self-reported psychiatric disease, regular medication use, and chronic disease were present in 24 (9.1%), 17 (6.4%), and 15 (5.7%) students, respectively. Overall, 133 students (50.2%) had poor sleep quality and 39 (14.7%) met the threshold for high NES risk (Table 1).

Sex comparisons showed several statistically significant differences (Table 2). Males had higher BMI, whereas females had higher PSQI, ESS scores, HAM-A, and emotional eating scores. Mean NEQ scores did not differ significantly by sex. Smoking was more frequent among males (16.8% vs. 4.6%; *p* = 0.002), whereas alcohol use did not differ significantly (11.5% vs. 8.6%; *p* = 0.555). Poor sleep quality (45.1% vs. 53.9%; *p* = 0.195) and high NES risk (18.6% vs. 11.8%; *p* = 0.175) did not differ significantly between sexes.

### 3.2. Sleep Quality and Night Eating Syndrome

Poor sleep quality (PSQI > 5) was observed in 133 students (50.2%); good sleep quality in 132 (49.8%). Mean PSQI was 5.71 ± 2.65. High NES risk (NEQ ≥ 25) was present in 39 students (14.7%). Mean NEQ was 17.03 ± 7.40. Poor sleep quality was numerically more frequent in females than males (53.9% vs. 45.1%), whereas high NES risk was numerically more common in males (18.6% vs. 11.8%); neither sex difference reached statistical significance (χ^2^*p* = 0.195 and *p* = 0.175, respectively; Table 2). Mean NEQ scores were significantly higher in the poor-sleep group than in the good-sleep group (Table 3).

### 3.3. Restless Legs Syndrome and Chronotype

The mean RLS symptom score was 3.24 ± 6.38. No symptoms (score 0) were reported by 173 students (65.3%); mild (1–10), moderate (11–20), and severe (21–30) symptoms were observed in 60 (22.6%), 21 (7.9%), and 11 (4.2%) participants, respectively, resulting in an overall RLS symptom prevalence of 34.7% (score > 0). The RLS score demonstrated a weak correlation with NEQ (r = 0.114; *p* = 0.063) and exhibited a modest correlation with PSQI (r = 0.141; *p* = 0.021).

Most participants exhibited an intermediate chronotype (239; 90.2%), with 20 (7.5%) identified as evening types and 6 (2.3%) as morning types. Females demonstrated higher MEQ scores compared to males (49.61 ± 5.24 versus 47.87 ± 5.26; *p* = 0.024), indicating a relative preference for morningness among females. The MEQ did not show a significant correlation with NEQ (r = 0.017; *p* = 0.789) or PSQI (r = 0.078; *p* = 0.208).

### 3.4. Psychiatric Comorbidity

Students who reported a known psychiatric diagnosis (*n* = 24) exhibited significantly higher scores on the PSQI (7.54 ± 2.89 versus 5.53 ± 2.55; *p* = 0.003), ISI (9.33 ± 4.42 versus 6.81 ± 3.72; *p* = 0.010), and HAM-A assessments (21.17 ± 10.21 versus 11.63 ± 8.45; *p* < 0.001) compared to those without such a diagnosis. The NEQ scores were numerically elevated in the group with psychiatric comorbidity (20.79 ± 9.12 versus 16.66 ± 7.08; *p* = 0.053).

### 3.5. Correlation Analysis

**Figure 1:**
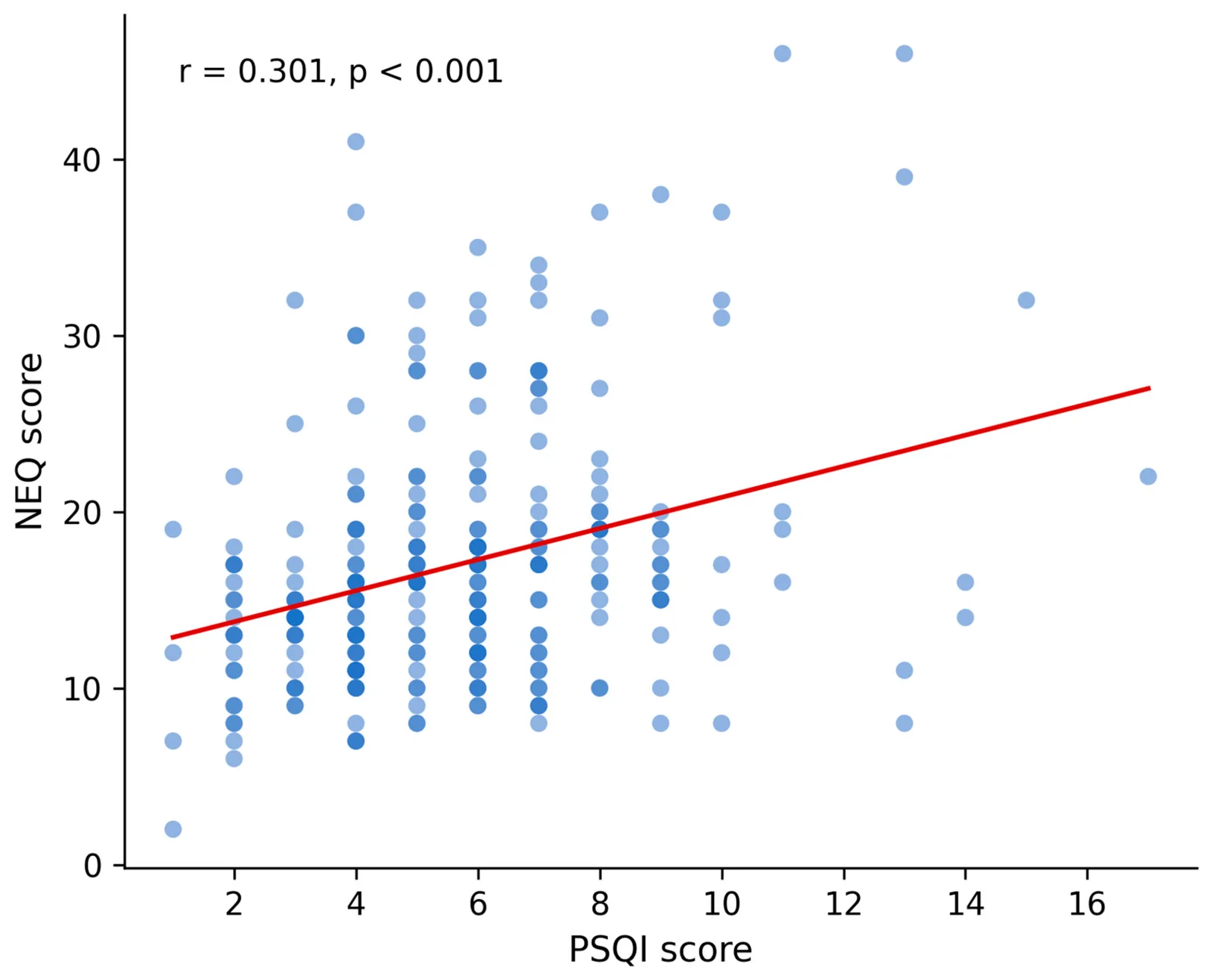
PSQI vs. NEQ correlation (r = 0.301; *p* < 0.001). Note: The solid line indicates the linear regression fit. Each point represents one participant. Points are displayed with partial transparency; darker blue tones indicate overlapping observations at similar PSQI-NEQ score combinations, not distinct subgroups.

**Figure 2:**
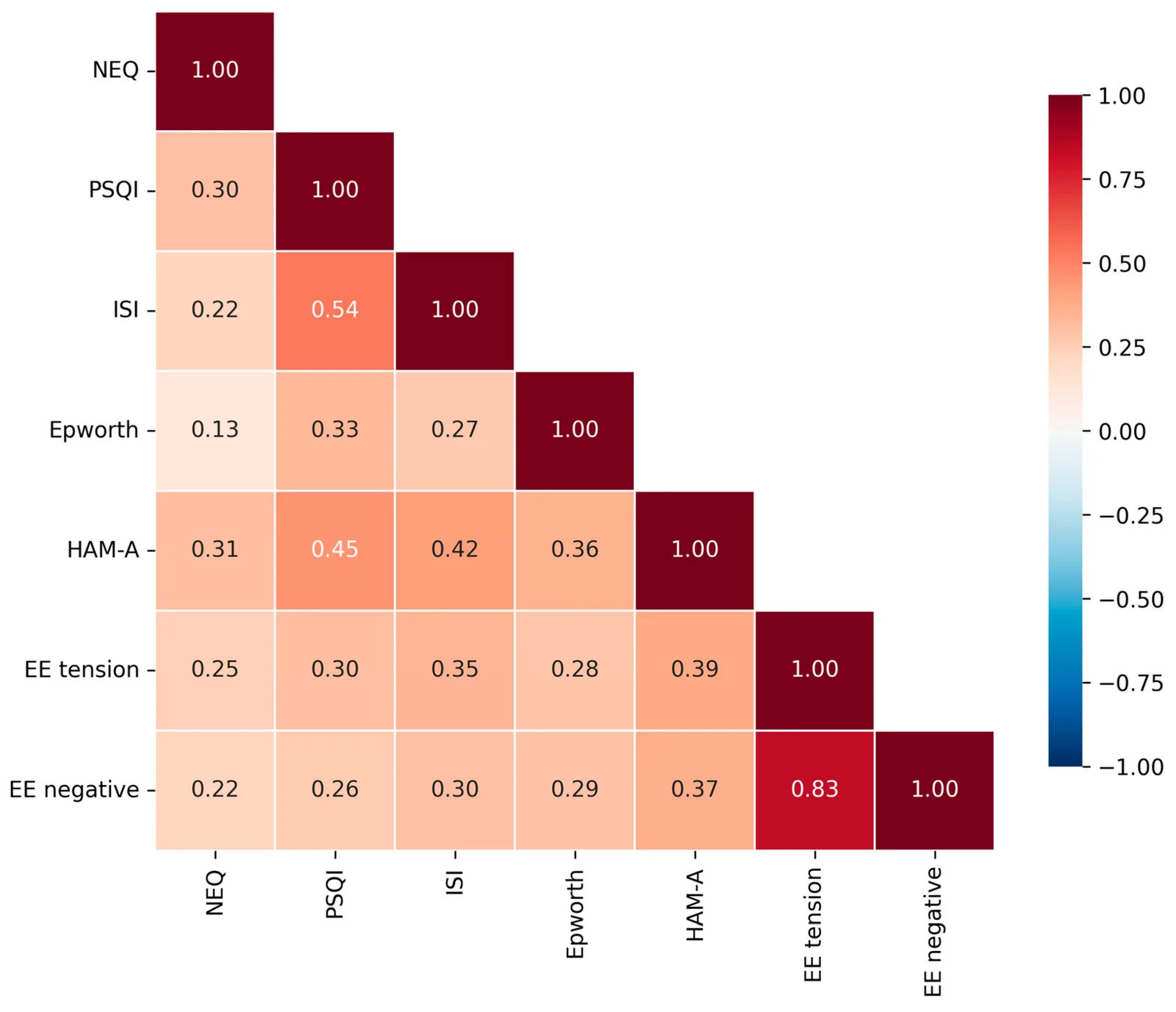
Correlation heatmap for key variables (coefficients shown in each cell).

The NEQ was significantly correlated with PSQI (r = 0.301; 95% CI [0.187, 0.407]; *p* < 0.001), HAM-A (r = 0.307; 95% CI [0.194, 0.413]; *p* < 0.001), ISI (r = 0.224; *p* < 0.001), emotional eating subscales (r = 0.222–0.246; *p* < 0.001), and the ESS score (r = 0.130; *p* = 0.035). The magnitudes of the NEQ–PSQI and NEQ–HAM-A correlations were similar, with overlapping confidence intervals. In bivariate analysis, smoking (r = 0.170; *p* = 0.006) and alcohol consumption (r = 0.166; *p* = 0.007) demonstrated positive associations with NEQ (Table 4). Furthermore, PSQI exhibited significant correlations with ISI (r = 0.538; *p* < 0.001), HAM-A (r = 0.446; *p* < 0.001), and emotional eating subscales (r = 0.260–0.305; *p* < 0.001). The PSQI–NEQ association is also illustrated in Figure 1; complete correlation results are shown in Table 5 and Figure 2.

### 3.6. Regression Analysis

In the primary multivariable model including only PSQI and sex, PSQI was the strongest independent correlate of NEQ scores (B = 0.91, standardized β = 0.325; *p* < 0.001), whereas sex was not statistically significant (B = −1.35, standardized β = −0.090; *p* = 0.126) (Table 6). Sensitivity analyses demonstrated that the PSQI coefficient remained materially stable after additional adjustment for smoking and alcohol use (standardized β = 0.294) and after further inclusion of age and BMI (standardized β = 0.291) (Table 7). By contrast, the sex coefficient was unstable across specifications, changing in magnitude and direction when age, BMI, smoking, and alcohol were added (B = −1.35 in the primary model versus B = −0.76 in the full model and B = 0.12 when PSQI was excluded), although it remained non-significant in all models (Table 7). The full multivariable model is shown in Table 8. In extended models, alcohol use was positively associated with NEQ (full model: B = 3.63, standardized β = 0.146; *p* = 0.024), whereas smoking was not significant (Table 8). In a secondary analysis excluding PSQI, alcohol use remained significant (B = 4.14, standardized β = 0.167; *p* = 0.014), while smoking showed a borderline association (B = 3.12, standardized β = 0.126; *p* = 0.068) (Table 9). VIF values in the full model exceeded 10 for age (51.5) and BMI (45.0), reflecting collinearity with sex in this sample; VIFs for sex (2.50), smoking (1.46), alcohol (1.39), and PSQI (5.98) were acceptable (Table 10). Age and BMI were retained in the full model only to report the complete prespecified covariate set. Because their VIF values exceeded 10, age and BMI coefficients in Table 8 are not interpreted inferentially; evaluation of the PSQI–NEQ association and sex coefficient stability rests on the primary and sensitivity models (Table 6 and Table 7).

## 4. Discussion

This cross-sectional study in Turkish medical students indicates that sleep disturbance and night-eating behaviors frequently co-occur in a population exposed to substantial academic stress. These patterns align with prior reports in university and medical student cohorts internationally [2,3,10,11,12,13,14,18], suggesting that sleep–eating links are not confined to clinical samples.

Sleep quality showed the most consistent association with night eating scores in both bivariate and multivariable analyses (Table 3, Table 6 and Table 7). The prespecified primary model—adjusting only for PSQI and sex—was chosen to limit multicollinearity and supports sleep disturbance as the principal correlate of NEQ in this sample. Sensitivity analyses indicated that the PSQI association was robust to additional adjustment, whereas sex was not a stable independent correlate across specifications. Anxiety and emotional eating clustered with sleep disturbance, consistent with shared stress-related pathways [6,9,35]. Because multiple correlation coefficients were examined without formal correction for multiple testing, bivariate associations should be viewed as exploratory; the multivariable framework nevertheless prioritizes the prespecified sleep–eating relationship. Causal direction cannot be inferred from this cross-sectional design.

Sex differences were evident for sleep, mood, and emotional eating burden, but not for mean NEQ scores or high NES screening status. Females reported greater sleep and psychological distress, whereas males showed numerically higher NES screening rates; these patterns mirror prior Turkish student data [12,16] and suggest that sex may modulate sleep and mood more than overt night eating screening scores in this setting.

Evening chronotype has been linked to night eating in some reports [17], but we found no NEQ–chronotype association, likely reflecting the predominantly intermediate chronotype distribution in our sample. Any tendency toward morningness in females should be interpreted cautiously given the narrow chronotype range.

RLS symptoms were common but were not specifically associated with night eating scores, implying partially overlapping yet distinct sleep-related pathways. RLS may contribute to global sleep disruption rather than NES-specific features in non-clinical students.

Positive associations between substance use and NEQ in exploratory analyses should be interpreted cautiously given binary assessment, low use prevalence, and unmeasured confounding. These findings highlight the need for validated substance-use measures in future work rather than supporting causal inference about nicotine or alcohol effects on night eating.

Self-reported psychiatric comorbidity was associated with broader sleep and anxiety burden and marginally higher night eating scores, supporting integrated mental health and sleep screening on campus [36].

Our findings support integrated campus initiatives addressing sleep hygiene, stress management, emotional eating, and comorbid sleep symptoms [15,16,37]. Students with elevated night eating and sleep disturbance scores may benefit from evaluation that addresses both domains rather than isolated disorder screening.

Strengths of this study include validated instruments, comprehensive assessment of sleep and eating behaviors, and focus on an underrepresented population. Limitations include the cross-sectional design, reliance on self-reported measures, and a single-center medical student sample. No formal a priori sample-size or power calculation was performed; the prespecified recruitment target of 300 participants was a feasibility-oriented goal, and findings should be interpreted with attention to estimate precision (*n* = 265). Psychiatric disorders, chronic illnesses, and medication use were captured only as binary self-reported variables without detailed diagnostic or pharmacological classification and were not verified against medical records. The Turkish NEQ was validated in psychiatric outpatients rather than non-clinical students [20], and the ≥25 threshold was applied as a screening cutpoint [10,11]; accordingly, findings reflect screening-based NES risk rather than clinical diagnosis. PSQI and NEQ share conceptual overlap regarding sleep-related and nocturnal eating features, which may inflate their association and motivated our secondary model excluding PSQI. In the full covariate model, VIF values exceeded 10 for age and BMI because of collinearity with sex; coefficients for age and BMI in Table 8 are therefore not interpreted; PSQI and sex associations were evaluated using the primary and sensitivity models (Table 6 and Table 7). Smoking and alcohol use were not quantified in terms of amount, frequency, or dependence severity. Unmeasured academic stressors—including examination periods, irregular schedules, and caffeine or energy consumption—were not systematically assessed and may confound sleep and eating outcomes. SRED screening items were included in the survey battery but were not analyzed in the final merged dataset; the study therefore primarily addresses NES screening with NEQ rather than SRED. Future prospective studies with clinical interview confirmation are recommended.

## 5. Conclusions

Poor sleep quality and high night eating syndrome (NES) screening risk are prevalent and cross-sectionally associated among Gülhane medical students. In the primary multivariable model, PSQI was the strongest independent correlate of NEQ scores. These findings support integrated screening for sleep disturbance, night eating behavior, anxiety, and comorbid sleep symptoms in Turkish medical education settings.

## Figures and Tables

**Table 1:** Sociodemographic and clinical characteristics (*n* = 265).

Variable	Value
Total participants, *n*	265
Female, *n* (%)	152 (57.4)
Male, *n* (%)	113 (42.6)
Age (years), mean ± SD	21.0 ± 1.7
BMI (kg/m^2^), mean ± SD	22.48 ± 3.30
Smoking, *n* (%)	26 (9.8)
Alcohol use, *n* (%)	26 (9.8)
Psychiatric disease, *n* (%)	24 (9.1)
Regular medication, *n* (%)	17 (6.4)
Chronic disease, *n* (%)	15 (5.7)
Poor sleep quality (PSQI > 5), *n* (%)	133 (50.2)
High NES risk (NEQ ≥ 25), *n* (%)	39 (14.7)

Note: Psychiatric disease, chronic disease, and regular medication use were assessed as binary self-reported variables without diagnostic subtyping or pharmacological classification; diagnoses were not verified against medical records. SD: Standard Deviation, BMI: Body-Mass Index, PSQI: Pittsburgh Sleep Quality Index, NES: Night Eating Syndrome, NEQ: Night Eating Questionnaire.

**Table 2:** Comparison of variables by sex.

Variable	Male	Female	*p*
Age (years)	21.67 ± 1.65	20.53 ± 1.59	<0.001
BMI (kg/m^2^)	24.25 ± 3.07	21.16 ± 2.82	<0.001
NEQ	17.50 ± 8.10	16.68 ± 6.84	0.603
PSQI	5.37 ± 2.79	5.96 ± 2.51	0.031
ISI	6.78 ± 4.04	7.24 ± 3.69	0.127
ESS	5.87 ± 3.74	7.26 ± 3.81	0.004
Emotional eating–tension	22.06 ± 7.50	26.24 ± 9.30	<0.001
Emotional eating–negative emotions	19.48 ± 7.56	22.60 ± 8.62	0.003
HAM-A	10.50 ± 8.14	13.99 ± 9.30	0.002
MEQ	47.87 ± 5.26	49.61 ± 5.24	0.024
RLS score	3.19 ± 5.48	3.27 ± 6.98	0.210
Smoking, *n* (%)	19 (16.8)	7 (4.6)	0.002
Alcohol, *n* (%)	13 (11.5)	13 (8.6)	0.555
High NES risk, *n* (%)	21 (18.6)	18 (11.8)	0.175
Poor sleep, *n* (%)	51 (45.1)	82 (53.9)	0.195

BMI: Body-Mass Index, NEQ: Night Eating Questionnaire, PSQI: Pittsburgh Sleep Quality Index, ISI: Insomnia Severity Index, ESS: Epworth Sleepiness Scale, HAM-A: Hamilton Anxiety Rating Scale, MEQ: Morningness–Eveningness Questionnaire, RLS: Restless Legs Syndrome, NES: Night Eating Syndrome.

**Table 3:** Comparison of NEQ scores by sleep quality group (*n* = 265). Comparisons were performed using the Mann–Whitney U test.

Sleep Quality Group	*n*	NEQ, Mean ± SD	*p*	Cohen’s d
Good sleep (PSQI ≤ 5)	132	15.38 ± 6.36		
Poor sleep (PSQI > 5)	133	18.67 ± 8.00	<0.001	0.455

PSQI: Pittsburgh Sleep Quality Index, NEQ: Night Eating Questionnaire.

**Table 4:** Bivariate Spearman’s correlations between NEQ and lifestyle or anthropometric variables (*n* = 265).

Variable	r	95% CI	*p*
Smoking (binary)	0.170	[0.050, 0.284]	0.006
Alcohol use (binary)	0.166	[0.047, 0.281]	0.007
Age (years)	0.091	[−0.030, 0.209]	0.139
BMI (kg/m^2^)	0.032	[−0.089, 0.152]	0.605

BMI: Body-Mass Index.

**Table 5:** Spearman’s correlations among key variables (*n* = 265).

Variable 1	Variable 2	r	95% CI	*p*
NEQ	PSQI	0.301	[0.187, 0.407]	<0.001
NEQ	ISI	0.224	[0.106, 0.335]	<0.001
NEQ	ESS	0.130	[0.009, 0.246]	0.035
NEQ	HAM-A	0.307	[0.194, 0.413]	<0.001
NEQ	Emotional eating–tension	0.246	[0.129, 0.356]	<0.001
NEQ	Emotional eating–negative emotions	0.222	[0.105, 0.334]	<0.001
PSQI	ISI	0.538	[0.446, 0.618]	<0.001
PSQI	ESS	0.335	[0.223, 0.438]	<0.001
PSQI	HAM-A	0.446	[0.344, 0.538]	<0.001
PSQI	Emotional eating–tension	0.305	[0.191, 0.410]	<0.001
PSQI	Emotional eating–negative emotions	0.260	[0.144, 0.369]	<0.001
ISI	Emotional eating–tension	0.345	[0.234, 0.447]	<0.001
ISI	Emotional eating–negative emotions	0.304	[0.190, 0.409]	<0.001
ESS	Emotional eating–tension	0.283	[0.168, 0.390]	<0.001
ESS	Emotional eating–negative emotions	0.294	[0.179, 0.400]	<0.001

PSQI: Pittsburgh Sleep Quality Index, NEQ: Night Eating Questionnaire, ISI: Insomnia Severity Index, ESS: Epworth Sleepiness Scale, HAM-A: Hamilton Anxiety Rating Scale.

**Table 6:** Primary multiple linear regression model for NEQ score (PSQI and sex; *n* = 265).

Independent Variable	B (95% CI)	Standardized β	*p*
Female sex	−1.35 (−3.07 to 0.38)	−0.090	0.126
PSQI	0.91 (0.59 to 1.23)	0.325	<0.001
Model summary			
R^2^	0.108		
Adjusted R^2^	0.101		
F (df)	15.79 (2, 262)		<0.001

PSQI: Pittsburgh Sleep Quality Index.

**Table 7:** Sensitivity analyses comparing PSQI and sex coefficients across regression specifications (*n* = 265).

Parameter	Model 1: PSQI + Sex	Model 2: +Smoking, Alcohol	Model 3: +Age, BMI (Full)	Model 4: Lifestyle Without PSQI
PSQI: B (β), *p*	0.91 (0.325), <0.001	0.82 (0.294), <0.001	0.81 (0.291), <0.001	—
Female sex: B (β), *p*	−1.35 (−0.090), 0.126	−0.91 (−0.061), 0.306	−0.76 (−0.051), 0.467	0.12 (0.008), 0.913
R^2^ (adjusted R^2^)	0.108 (0.101)	0.148 (0.135)	0.148 (0.129)	0.068 (0.050)
F, *p*	15.79, <0.001	11.29, <0.001	7.49, <0.001	3.78, 0.003

PSQI: Pittsburgh Sleep Quality Index.

**Table 8:** Full multiple linear regression model for NEQ score (*n* = 265).

Independent Variable	B (95% CI)	Standardized β	*p*
Female sex	−0.76 (−2.81 to 1.29)	−0.051	0.467
Age (years)	0.08 (−0.44 to 0.61)	0.019	0.753
BMI (kg/m^2^)	0.02 (−0.27 to 0.30)	0.007	0.912
Smoking (yes)	2.29 (−0.93 to 5.52)	0.092	0.162
Alcohol use (yes)	3.63 (0.48 to 6.77)	0.146	0.024
PSQI	0.81 (0.49 to 1.14)	0.291	<0.001
Model summary			
R^2^	0.148		
Adjusted R^2^	0.129		
F (df)	7.49 (6, 258)		<0.001

BMI: Body-Mass Index, PSQI: Pittsburgh Sleep Quality Index.

**Table 9:** Secondary multiple linear regression model for NEQ score excluding PSQI (*n* = 265).

Independent Variable	B (95% CI)	Standardized β	*p*
Female sex	0.12 (−1.99 to 2.23)	0.008	0.913
Age (years)	0.22 (−0.32 to 0.77)	0.051	0.421
BMI (kg/m^2^)	0.06 (−0.24 to 0.35)	0.025	0.716
Smoking (yes)	3.12 (−0.23 to 6.47)	0.126	0.068
Alcohol use (yes)	4.14 (0.86 to 7.43)	0.167	0.014
Model summary			
R^2^	0.068		
Adjusted R^2^	0.050		
F (df)	3.78 (5, 259)		0.003

BMI: Body-Mass Index.

**Table 10:** Variance inflation factors (VIFs) for the full multivariable regression model (*n* = 265).

Independent Variable	VIF
Female sex	2.50
Age (years)	51.54
BMI (kg/m^2^)	44.98
Smoking (yes)	1.46
Alcohol use (yes)	1.39
PSQI	5.98

BMI: Body-Mass Index, PSQI: Pittsburgh Sleep Quality Index.

## Data Availability

The original contributions presented in this study are included in the article material. Further inquiries can be directed to the corresponding author.

## Supplementary Materials

The following supporting information can be downloaded at: https://www.mdpi.com/article/10.3390/healthcare14162666/s1, Table S1: STROBE checklist for cross-sectional studies.
