# Supplementary material for: Association of Night Eating Syndrome Risk with Sleep Disturbance in Medical Students: A Cross-Sectional Study
Source: Healthcare (Basel). 2026 Aug 21;14(16):2666. doi: 10.3390/healthcare14162666 (PMC13512618; doi:10.3390/healthcare14162666)
Supplement: Supplementary file 1 [file healthcare-14-02666-s001.zip › healthcare-4474512-supplementary.pdf]

Table S1. STROBE Checklist for Cross-Sectional Studies

Manuscript: Association of Night Eating Syndrome Risk with Sleep Disturbance in Medical Students: A Cross-Sectional Study

Authors: Volkan Tekin et al.

Checklist version: STROBE statement—cross-sectional studies (v. 4)

Date completed: 9 July 2026

Section	Item	Checklist recommendation	Page(s)	Reported	Notes
Title and abstract	1a	Indicate the study design with a commonly used term in the title or the abstract	Title, p. 1; Abstract, p. 1	Yes	Title includes “Cross-Sectional Study”; Abstract Methods states cross-sectional design.
Title and abstract	1b	Provide in the abstract an informative and balanced summary of what was done and what was found	Abstract, p. 1	Yes	Structured abstract covers background, objective, methods, results, conclusions.
Introduction	2	Explain the scientific background and rationale for the investigation being reported	Introduction, pp. 1–2	Yes	Background on sleep–eating disorders, student vulnerability, Turkish literature gap.
Introduction	3	State specific objectives, including any prespecified hypotheses	Introduction, p. 2; Abstract, p. 1	Yes	Prevalence estimation and cross-sectional association objectives stated; no formal a priori hypotheses.
Methods	4	Present key elements of study design early in the paper	Methods, pp. 2–3	Yes	Study Design and Setting section specifies cross-sectional observational design.
Methods	5	Describe the setting, locations, and relevant dates	Methods, p. 2	Yes	Gülhane Faculty of Medicine, Ankara, Türkiye; 1 March–31 December 2023.

Methods	6a	Give the eligibility criteria, and the sources and methods of selection of participants	Methods, p. 2	Yes	Inclusion/exclusion criteria; voluntary recruitment via classroom announcements and electronic survey.
Methods	6b	For matched studies, give matching criteria and number of exposed and unexposed	—	N/A	Not a matched design.
Methods	7	Clearly define all outcomes, exposures, predictors, potential confounders, and effect modifiers	Methods, pp. 2–3; Results, pp. 4–11	Yes	NEQ (outcome), PSQI and lifestyle variables (exposures/predictors), cut-offs defined (PSQI >5, NEQ ≥25).
Methods	8	For each variable of interest, give sources of data and details of methods of assessment (measurement)	Methods, pp. 2–3	Yes	Validated instruments listed with references (NEQ, PSQI/PUKI, ISI, Epworth, HAM-A, emotional eating, RLS, MEQ).
Methods	9	Describe any efforts to address potential sources of bias	Methods, p. 3; Discussion, p. 12	Partial	Structured HAM-A interview and validated tools reduce bias; no formal bias analysis. Limitations discuss self-report and confounding.
Methods	10	Explain how the study size was arrived at	Methods, p. 2	Partial	Ethics protocol prespecified target n≥300; response rate reported (265/300). Formal power/sample-size calculation not provided.
Methods	11	Explain how quantitative variables were handled in the analyses	Methods, p. 3	Yes	Continuous variables as mean±SD; normality assessed (Shapiro–Wilk); non-parametric tests when appropriate; categorical cut-offs prespecified.
Methods	12a	Describe all statistical	Methods, p. 3	Yes	Mann–Whitney U, chi-

		methods, including those used to control for confounding			square, Spearman correlation; multiple linear regression with covariates; VIF for multicollinearity.
Methods	12b	Describe any methods used to examine subgroups and interactions	Results, pp. 4–11	Partial	Sex-stratified comparisons and psychiatric comorbidity subgroup reported; formal interaction tests not performed.
Methods	12c	Explain how missing data were addressed	Methods, p. 2	Partial	Participants with incomplete questionnaires excluded (n=35); item-level missing-data handling not described.
Methods	12d	If applicable, describe any cohort profile and loss to follow-up	—	N/A	Cross-sectional single-contact survey; no follow-up.
Methods	12e	Describe any sensitivity analyses	Methods, p. 3; Results, pp. 10–11	Yes	Secondary regression model excluding PSQI to reduce construct overlap; multiple-comparison caution noted for correlation matrix.
Results	13a	Report numbers of individuals at each stage of study—e.g., numbers potentially eligible, examined for eligibility, confirmed eligible, included in the study, completing follow-up, and analyzed	Methods, p. 2; Results, p. 4	Yes	300 approached/self-enrolled; 265 analyzed; 35 excluded (incomplete questionnaires or consent withdrawal).
Results	13b	Give reasons for non-participation at each stage	Methods, p. 2	Yes	Exclusion due to incomplete questionnaires or withdrawal of consent.
Results	14a	Give characteristics of study participants (e.g., demographic, clinical,	Results, pp. 4–6; Tables 1–2	Yes	Table 1 sociodemographics; Table 2 sex comparisons across

		social) and information on exposures and potential confounders			all key variables.
Results	14b	Indicate number of participants with missing data for each variable of interest	—	No	Not reported by variable; analysis restricted to complete cases (n=265).
Results	15	Report numbers of outcome events or summary measures	Results, pp. 4–11	Yes	Prevalence counts (poor sleep 50.2%, high NES risk 14.7%, RLS symptoms 34.7%); means, correlations, regression coefficients reported.
Results	16a	Give unadjusted estimates and, if applicable, confounder-adjusted estimates and their precision (e.g., 95% confidence interval). Make clear what confounders were adjusted for and why they were included	Results, pp. 8–11; Tables 3–6	Yes	Spearman r (unadjusted); regression B, standardized β, 95% CI, p (adjusted for sex, age, BMI, smoking, alcohol, PSQI).
Results	16b	Report category boundaries when continuous variables were categorized	Methods, pp. 2–3; Results, pp. 4–5	Yes	PSQI >5, NEQ ≥25, RLS 0/1–10/11–20/21–30, MEQ chronotype categories defined.
Results	16c	If relevant, consider translating estimates of relative risk into absolute risk for a meaningful time period	—	N/A	Linear/correlational estimates; not applicable for absolute risk translation.
Results	17	Report other analyses done—e.g., analyses of subgroups and interactions, and sensitivity analyses	Results, pp. 6–11	Yes	Sex comparisons, psychiatric comorbidity subgroup, secondary regression without PSQI, VIF table.
Discussion	18	Summarize key results with reference to study objectives	Discussion, pp. 12–13	Yes	Prevalence, PSQI–NEQ association, regression findings linked to

					objectives.
Discussion	19	Discuss limitations of the study, taking into account sources of potential bias or imprecision	Discussion, p. 12	Yes	Cross-sectional design, self-report, single center, screening not diagnostic NES, multiple comparisons, SRED not analyzed, residual confounding.
Discussion	20	Give a cautious overall interpretation of results considering objectives, limitations, multiplicity of analyses, and similar studies	Discussion, pp. 12–13	Yes	Causal language avoided; unexpected alcohol finding interpreted cautiously; MEQ finding qualified.
Discussion	21	Discuss the generalizability (external validity) of the study results	Discussion, pp. 12–13	Partial	Turkish single-center medical students; generalizability implied but not discussed in dedicated paragraph.
Other information	22	Give the source of funding and the role of the funders for the present study	Declarations, p. 13	Yes	Funding: No funding. Conflict of interest declared: none.

Summary of reporting completeness:

Fully reported (Yes): 26 items | **Partially reported:** 5 items (9, 10, 12b, 12c, 21) | **Not reported:** 1 item (14b) | **Not applicable:** 3 items (6b, 12d, 16c)

Reference: von Elm E, Altman DG, Egger M, Pocock SJ, Gøtzsche PC, Vandenbroucke JP. The Strengthening the Reporting of Observational Studies in Epidemiology (STROBE) statement: guidelines for reporting observational studies. Lancet. 2007;370(9596):1453-1457. STROBE cross-sectional checklist available at https://www.strobe-statement.org/checklists/.
